# Elevated Indoor Volatile Organic Compound Exposure in the Niger Delta Region of Nigeria

**DOI:** 10.3390/ijerph15091939

**Published:** 2018-09-06

**Authors:** Kalé Z. Kponee, Jamaji C. Nwanaji-Enwerem, Xianqiang Fu, Iyenemi I. Kakulu, Marc G. Weisskopf, Chunrong Jia

**Affiliations:** 1 Departments of Environmental Health and Epidemiology, Harvard T.H. Chan School of Public Health, Boston, MA 02115, USA; mweissko@hsph.harvard.edu; 2 Department of Environmental Health, Harvard T.H. Chan School of Public Health and MD-PhD Program, Harvard Medical School, Boston, MA 02115, USA; jamaji_Nwanaji-Enwerem@hms.harvard.edu; 3 School of Public Health, University of Memphis, Memphis, TN 38152, USA; xfu@memphis.edu (X.F.); cjia@memphis.edu (C.J.); 4 Department of Estate Management, Rivers State University, Port Harcourt, Rivers State PMB 5080, Nigeria; ibkakulu@hotmail.com

**Keywords:** volatile organic compounds, indoor air quality, public health, cancer risk, Niger Delta, oil production

## Abstract

The implications of environmental contamination on human health in the Niger Delta region of Nigeria remain a topic of growing international public health interest. To better understand ongoing air pollution and initiate remediation efforts, the United Nations Environmental Programme (UNEP) report recommended the monitoring of volatile organic compounds (VOCs) across different media (water, soil, and air) in Ogoniland, an at-risk population in the Niger Delta region of Nigeria. In this pilot study, we measured indoor VOC concentrations in the indoor air of 20 households in Ogale, an Ogoniland community whose groundwater system is contaminated with benzene at levels 900 times the World Health Organization guidelines and evaluated self-reported health conditions and predicted cancer risks and hazards from inhalation exposure to VOCs. We detected higher concentrations of benzene (mean = 25.7 μg/m^3^, SD = 23.2 μg/m^3^) and naphthalene (mean = 7.6 μg/m^3^, SD = 13.8 μg/m^3^) than has been reported in other regions. Although study participants reported health symptoms consistent with VOC exposure, we were underpowered to detect a significant association between select indoor VOCs and these self-reported health symptoms using univariate logistic regression models. These findings suggest that that the health symptoms reported by participants may be poor proxies for the underlying disease processes associated with adverse health outcomes due to VOC exposure in this community and that the burden of adverse health effects due to VOC exposure may stem from the contaminated groundwater system. We estimated a non-cancer hazard quotient of 3 from exposure to naphthalene and lifetime excess cancer risks from exposure to naphthalene, benzene, p-dichlorobenzene, carbon tetrachloride, and ethylbenzene of 3 × 10^−4^, 2 × 10^−4^, 6 × 10^−5^, 6 × 10^−6^, and 1 × 10^−5^, respectively. These results exceed common risk benchmarks in the United States, suggesting a need for further studies to characterize VOC exposures, sources, and associated health risks in the Niger Delta.

## 1. Introduction

The implications of environmental contamination on human health in the Niger Delta region of Nigeria have been a topic of growing international public health interest [1]. Since the discovery of commercial oil quantities in the region in the 1950s, many of its communities have been subject to ambient chemical exposures from venting, flaring, and other activities of oil production [2]. The United States Department of Energy estimates that since the 1960s, well over 4000 oil spills—involving emissions of millions of barrels of crude oil—have occurred in the Niger Delta [3]. A recent study reported that Niger Delta residents have sustained additional health risks due to numerous oil-related accidents [4]. Moreover, the limited efforts for environmental clean-up and remediation following these accidents may confer additional health risks on residents in the region [1]. Exposures to volatile organic compounds (VOCs) have been highlighted as one of the long-lasting, major ambient chemical risks associated with oil production and accidents in the Niger Delta. In a 2011 report focusing on Ogoniland, a region in the Niger Delta affected by oil-related contamination, the United Nations Environmental Programme (UNEP) reported the detection of benzene—a known carcinogenic VOC. Specifically, concentrations of benzene in sampling locations across Ogoniland ranged from 0.2 to 48.2 µg/m^3^. To better understand ongoing air pollution and monitor remediation, the UNEP report recommended the monitoring of VOCs across different media (water, soil, and air) in Ogoniland [5]. 

To date, a limited number of air quality studies have been conducted in the Niger Delta [2,6]. However, these studies may not offer a complete picture regarding the exposures and associated health risks of Ogoni residents. Several studies conducted in the Niger Delta have relied on qualitative methods to ascertain indoor air quality or have focused on outdoor air quality [2,6,7,8]. To our knowledge, no studies have measured indoor VOC levels in Ogoniland or the Niger Delta, and estimated health risks associated with the indoor exposure. In this pilot study, we measured VOC concentrations in 20 homes across Ogale, a community in the Eleme local government area of Ogoniland identified as having the most serious refined oil contamination in Ogoniland [5]. In Ogale, UNEP monitoring detected benzene and naphthalene in individual borehole drinking water wells with benzene concentrations as high as 9280 µg/L [5]. Additionally, a 2015 pilot study found higher risks of self-reported symptoms consistent with exposure to petroleum contaminated water in Ogale when compared to a similar, non-exposed community [9]. We estimated the cancer risks and non-cancer hazards associated with VOC exposure, and investigated self-reported health symptoms and conditions. Through this indoor air quality study and risk assessment study, we make early efforts to gain a broader understanding of total VOC exposures and their potential impacts on health in Ogoniland and the Niger Delta region of Nigeria. 

## 2. Methods

### 2.1. Study Population

The people of Ogale are from the local government area of Eleme, in Ogoniland. We collaborated with Rivers State University (RSU) and community leaders to recruit Ogale residents for the study. Participants were selected from a convenience sample of residents recruited by community leaders from different areas in Ogale. A total of 20 adults over the age of 18 from distinct households were enrolled. Participants met the following eligibility criteria: (1) residence in the Ogale community for a minimum of three years since study recruitment; and (2) no prior history of residence in any other Ogoniland community associated with high levels of petroleum contamination, as reported by UNEP [5]. This study was conducted in accordance with the Declaration of Helsinki, and with approval from local authorities in Ogale, the Joint College Ethics Committee at RSU, and from the Institutional Review Board (IRB) at the Harvard T.H Chan School of Public Health. Informed consent was obtained for each participant. The protocol was approved by the IRB at the Harvard T.H. Chan School of Public Health (Project identification code: IRB16-1928) on 19 December 2016. 

### 2.2. VOC Monitoring

VOC monitoring occurred during Nigeria’s dry season in the month of January 2017. VOCs in indoor and outdoor air samples were collected in a passive way using standard ATD type tubes packed with Tenax TA (Model: C1-AXXX-5003, Markes International Inc., Llantrisant, UK). This passive sampling method has been validated and applied in many studies [10,11,12,13]. Based on the tube configuration, the sampling rate was 0.16–0.43 mL/min, depending on the compound. Passive sampling tubes were placed in a central location above the ground, and away from windows, doors, and supplies in each participant’s household for a 48-h period. Simultaneous outdoor samples were collected at two locations, assuming that outdoor VOC concentrations are less variable in a small area. After sampling, tubes were sealed with storage caps, wrapped in pre-cleaned aluminum foil, and stored in a sealed stainless-steel jar at room temperature. All sampling occurred in January 2017; the average indoor temperature at sampling tube deployment was 33.7 °C and the average outdoor temperature was 32.7 °C during sampling periods. 

All samples were stored in a cool location until they were shipped to an analytical chemistry laboratory at the University of Memphis in Memphis, Tennessee, for analysis. All samples were analyzed within 20 days after field collection following well-developed procedures [14]. The tubes were analyzed on an automated thermal desorption (ULTRA 2 + UNITY 2, Markes International, Llantrisant, UK)—gas chromatography/mass spectrometry (Agilent 7890A/5975C, Agilent, Santa Clara, CA, USA) (TD-GC/MS) system following well-developed programs [15]. The GC was equipped with a HP-5MSUI column (30 m × 250 µm × 0.25 µm), the MS was operated in a scan mode, and the system was calibrated for 70 target VOCs. After instrumental analysis, the target compounds were identified and confirmed using NIST 2005 Spectral Library in ChemStation [15] The method detection limits ranged from 0.10 to 0.67 µg/m^3^ based on two-day passive sampling. No VOCs were detected in blank tubes and the duplicate precision averaged 2.8%. Measures of central tendency and estimates of variability were calculated for each VOC. The concentration of each VOC was summed, and the total VOC concentration was calculated as a summary measure.

### 2.3. Questionnaire

Two trained interviewers, one from the Harvard T.H Chan School of Public of Health in Massachusetts and another from RSUST in Rivers State, administered standardized questionnaires in each participant’s home. We used an amended version of the questionnaires developed by Kponee et al. for their pilot study in the same community [9]. We asked primarily closed-ended questions regarding demographics and smoking habits, and open-ended questions regarding current health symptoms and medical history. Participants who reported currently experiencing health symptoms were asked to list their symptoms. Their responses were subsequently grouped by symptom type (neurologic, skin irritation, throat irritation, and gastrointestinal). Participants were asked if they had ever been diagnosed with leukemia or aplastic anemia as these health conditions are consistent with benzene exposure. 

### 2.4. Health Risk Assessment and Adverse Health Symptoms

We conducted a screening level risk assessment to prioritize VOCs. We evaluated the risk of chronic inhalation non-cancer effects by comparing measured VOC concentrations with reference concentrations (RfCs) from U.S. EPA’s Integrated Risk Information System (IRIS), assuming that the current VOC measurements could represent long-term exposure [16]. Hazard quotients (HQs) were calculated by dividing the measured concentration of a VOC by its corresponding RfCs when available. Information on the physiological system on which the RfC is based is also provided in IRIS.

VOC inhalation cancer risks for individual carcinogenic compounds were estimated by multiplying the measured VOC concentration by its corresponding Inhalation Unit Risk (IUR; per µg/m^3^), from IRIS and California EPA, as summarized by US EPA [16,17]. Estimated cancer risks greater than 1 × 10^−6^, the benchmark risk value, may warrant concern. All analyses were performed using SAS 9.4 (SAS Institute, Cary, NC, USA). 

The numbers of patients in the study population experiencing health symptoms were expressed as proportions. Given the limited sample size, univariate logistic regression models were used to estimate the association between VOCs evaluated for chronic inhalation non-cancer effects and cancer risks, and self-reported health symptoms. 

## 3. Results 

### 3.1. VOC Exposure and Risk Estimates

A total of 19 VOCs were detected in indoor air of the 20 households (Table 1). All but two of the VOCs were detected in 100% of the samples, i.e., their detection frequencies (DF) were 100%. For 1,4-dioxane, the DF was 95%, and it was 50% for 2,5-dimethylfuran (DMF). Aromatic compounds such as benzene, toluene, ethylbenzene, xylenes (BTEX), and naphthalene had the highest indoor concentrations, with benzene having a maximum indoor concentration level of 105.4 µg/m^3^ (Table 2). VOC monitoring occurred for outdoor locations near two of the homes with indoor measurements in Ogale. In one home, the I/O ratios for each individual chemical ranged from 0.87 to 1.4, except for naphthalene (I/O = 3.3); in the other home, the I/O ratios ranged from 0.61 to 2.8, except for 2,5-DMF (I/O = 0) and *d*-limonene (I/O = 10.6).

Of the 19 detected VOCs, 10 have non-cancer health guidelines available in IRIS and the majority of these (60%) were based on neurological outcomes. Out of these 10 VOCs, only naphthalene (HQ = 3) had an HQ greater than 1.0. Benzene (HQ = 0.9) and xylenes (HQ = 0.1) had the next highest HQs (Table 2). The other VOCs all had HQs less than 0.01, and cumulatively, the sum of these small HQs contributed to less than 1% of the total HQs, i.e., the hazard index (HI). It should also be noted that 30% and 45% of homes had benzene and naphthalene concentrations exceeding the corresponding RfCs, respectively. Five of the 19 VOCs detected had estimated elevated lifetime cancer risks greater than the benchmark risk value of 1 × 10^−6^. Naphthalene had the greatest estimated lifetime cancer risk, followed by benzene, p-dichlorobenzene, ethyl benzene, and carbon tetrachloride (Table 2). 

### 3.2. Non-Cancer Health Outcomes

**Figure 1:**
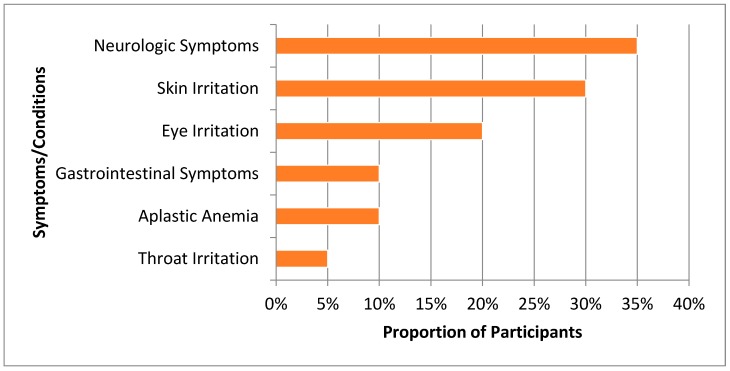
Adverse health symptoms and conditions reported by Ogale residents.

When asked about their current health symptoms or conditions, 35% (*n* = 7) of study participants reported having neurologic symptoms such as headaches, dizziness, sleepiness, and confusion (Figure 1). Among reports of irritations related to the body, skin irritation had the highest prevalence (30%, *n* = 6), followed by eye and throat irritation (20% (*n* = 4) and 5% (*n* = 1), respectively). Approximately 10% (*n* = 2) of study participants reported having gastrointestinal symptoms and 10% (*n* = 2) had diagnoses of aplastic anemia. None of the participants in our study reported having any respiratory symptoms or being diagnosed with leukemia. Table 3 shows the relationship between select indoor VOC concentrations and self-reported health symptoms. There were no significant associations between any of the indoor VOC concentrations evaluated and self-reported health symptoms. 

## 4. Discussion 

To our knowledge, this cross-sectional pilot study of indoor air quality is the first attempt to quantitatively characterize indoor VOC exposure and the associated health risks in the Niger Delta region of Nigeria. 

### 4.1. Indoor Air Pollution Sources for VOCs Posing Cancer and Non-Cancer Health Risks

While benzene levels in outdoor air locations sampled by UNEP ranged from 0.155 to 48.2 μg/m^3^ in Ogoniland [5], indoor benzene levels in our study ranged from 9.11 to 105.382 μg/m^3^ in Ogale. The architecture of Ogale homes is generally very open and so permits the free flow of indoor and outdoor air, suggesting that indoor air concentrations in Ogale might come from outdoor sources. In Ogale, benzene and naphthalene were detected in individual borehole drinking water wells with benzene concentrations approximately 1800 times the USEPA drinking water standard and 900 times the World Health Organization drinking water guidelines [5,9]. It is likely that the indoor air concentrations observed are driven in some part by the contamination in the groundwater system in Ogale. Historically, naphthalene is known for being a constituent of mothballs and tobacco smoke, while benzene is a known byproduct of oil/petroleum refining and can be produced from indoor sources like furniture, paints and adhesives, and cigarette smoke [18,19]. The VOC 2.5-Dimethylfuran is a tracer for tobacco smoke; however, it also exists in the smoke from wood burning [20]. Cooking stoves have been identified as an important indoor air pollution source in developing countries [21]. Thus, the detection of 2,5-DMF may indicate indoor wood burning for cooking. Particulate pollution from indoor wood burning may pose additional risks other than those conferred from outdoor VOCs. 

Limited outdoor monitoring suggested that outdoor VOCs significantly contributed to indoor VOCs. In one home, the I/O ratios for each individual chemical ranged from 0.87 to 1.4, except for naphthalene (I/O = 3.3), indicating outdoor VOCs as the dominant source. In the other home, the I/O ratios ranged from 0.61 to 2.8, except for 2,5-DMF (I/O = 0) and d-limonene (I/O = 10.6), indicating both indoor and outdoor contributions. In this present study, we were unable to assess sources of VOC exposure that contributed to I/O ratios greater than one. Given the ubiquitous petroleum hydrocarbon contamination in Ogoniland, it is possible that there are important outdoor sources of VOCs that drive indoor VOC concentrations in these homes. Systematic indoor and outdoor sampling is warranted to fully characterize indoor and outdoor VOC concentrations in Ogale, and to identify their sources. 

We observed differences in VOC concentrations in Ogale and other regions identified by our literature review. It was notable that our study detected one of the highest indoor benzene and naphthalene concentrations. The reasons for the differences observed may not be fully understood, but they likely involve regional differences in VOC concentrations, although differences in sampling methods, sampling periods, climate, human behaviors, and housing construction should also be considered [22]. In comparison to other areas of the world, fewer air pollution studies are focused on communities in sub-Saharan Africa [23]. Studies that explicitly measure the quality of air and examine any associated health risks in unique African environments will be critical for informing interventions tailored to the needs of those communities. 

### 4.2. Health Risks and Concerns

Consistent with the Kponee et al. study [9], Ogale participants in our study most frequently reported health symptoms consistent with central nervous system toxicity from exposure to VOCs, including headaches, confusion, and dizziness. Participants in Ogale also reported skin irritation, throat irritation, and a rash; symptoms consistent with VOC exposure [9,24,25]. In addition, 10% of participants reported experiencing aplastic anemia, another known endpoint of exposure to benzene and naphthalene [9,26]. Unlike previous studies in the Niger Delta and other studies conducted in occupational settings, participants in Ogale did not report respiratory symptoms typically associated with VOC exposure [9,27,28,29]. 

Our risk assessment analyses suggest that naphthalene poses the greatest predicted inhalation non-cancer and cancer risk to Ogale residents. Naphthalene, a low molecular weight VOC and polycyclic aromatic hydrocarbon, has been shown to adversely affect the hematopoietic system, damage red blood cells, and cause symptoms such as shortness of breath, confusion, and fatigue [30,31]. Animal studies have demonstrated that naphthalene also largely affects the nervous and respiratory systems [18]. Our risk assessment results indicate that exposure to naphthalene may be a health concern in Ogale. 

Given the larger body of literature surrounding outdoor air pollution, oil spillages, and VOCs, benzene has long been considered a major health risk for residents of the Niger Delta [32]. Additionally, the elevated range of indoor benzene concentrations found in the study suggests that Ogale participants may have a higher health risk profile than previously suggested. Benzene is a known carcinogen and is strongly associated with disorders of the immune and hematopoietic systems [19,26]. Given the elevated levels of benzene and the associated predicted cancer health risks, exposure to benzene poses health risks in Ogale. Cancer risks associated with naphthalene and benzene exposure exceeded 1 in 10,000 persons, a risk level that requires taking actions. The cumulative chemical risk from all these carcinogenic chemicals was 5 × 10^−4^, and naphthalene and benzene contributed 48% and 38%, respectively. These two aromatic compounds should be considered air toxics “risk drivers” in this community.

In addition to naphthalene and benzene, we found that exposure to p-dichlorobenzene, ethylbenzene, and carbon tetrachloride was associated with cancer risk estimates that warrant concern and public health action. Animal studies suggest that the inhalation or digestion of p-dichlorobenzene causes harmful liver effects [33]. In humans, inhalation of several p-dichlorobenzene isomers is associated with eye and nose irritation, dizziness, headaches, liver problems, and decreases in lung function [34]. Short-term exposure to ethylbenzene in the air has been associated with eye and throat irritation, vertigo, and dizziness [35]. In animal studies, long-term exposure from inhalation of ethylbenzene is associated with irreversible inner ear damage, kidney damage, and cancer that may also be relevant for human populations [35]. Although the effects of long-term exposure to low levels of carbon tetrachloride are relatively unknown, acute, elevated exposure to carbon tetrachloride has been associated with kidney and liver damage [36]. Other acute effects of exposure to carbon tetrachloride include neurological effects that present as headache, dizziness, sleepiness, nausea, and vomiting. The chronic and acute effects of carbon tetrachloride exposure can be reversed if exposure to the compound is eliminated [36]. Future studies should use biomonitoring methods to better assess exposure-related health effects of these VOCs in Ogale. 

Using univariate logistic regression models, we found no associations between indoor VOCs evaluated for non-cancer health effects and cancer risks, and self-reported health symptoms. There are several possible explanations for our findings. One explanation is that the health symptoms reported by study participants are poor proxies for the underlying disease processes associated with adverse health outcomes attributable to VOC exposure in this community. Additionally, these findings suggest that the critical source of exposure to VOCs in this population may not be through indoor air, but through exposure to the contaminated groundwater system in Ogale. In our study population, the average number of years subjects had lived in their current home was 23.2 years and the minimum and maximum number of years lived in their current home was four and 58 years, respectively. Given these figures, participants in Ogale are expected to be chronically exposed to VOCs via indoor air and via their groundwater system and may still utilize the contaminated water for household activities and drinking. It is possible that the etiology of self-reported symptoms in this community stems from the elevated VOC concentrations in the groundwater systems and not from VOC concentrations in indoor air. A third explanation for our finding is that our sample size of 20 participants limits our ability to detect meaningful associations between indoor VOC concentrations and self-reported health symptoms. Finally, given our limited sample size, we were unable to adjust for meaningful confounders such as age, sex, exposure to particulates, occupation, pre-existing health conditions, and other relevant characteristics. Univariate logistic regression models may be insufficient to elucidate the true relationship between indoor VOC concentrations and self-reported health symptoms in this community. Subsequent studies in this community should utilize larger sample sizes, assess the relationship between VOC concentrations in ground water and adverse health outcomes, and evaluate adverse health outcomes using more objective methods for disease assessment (e.g. biomarkers) to evaluate pathogenic processes resulting from chronic VOC exposure. 

### 4.3. Limitations

Although we found non-cancer and cancer health risks that suggest a need for further investigations, our study is not without limitations. First, this is a pilot study with a small sample size; second, a limited period of passive indoor air sampling precludes our ability to assess seasonal variability in VOC concentrations or long-term exposure. Third, we did not evaluate the sources of indoor VOCs; this makes it difficult to ascertain whether important infrastructures in the community such as an oil refinery, a major road, or use of household items associated with VOC exposure contribute to health risks observed in Ogale. We also identified indoor wood burning as a risk factor for adverse health, but did not measure particles or particle-bound chemicals such as polycyclic aromatic hydrocarbons (PAHs). Because participants in Ogale were recruited via convenience sampling, our results may not be representative of the entire community. To minimize the misclassification of self-report symptoms and health conditions, participants who reported experiencing any current health symptoms/conditions were asked to describe those symptoms to the interviewer. Furthermore, the cross-sectional nature of this risk assessment study and the insufficient adjustment for possible confounding variables, limits our ability to make causal inferences regarding the association between VOC concentrations and adverse health risks. All these limitations warrant larger follow-up studies that can assess the etiologic relationship between VOC concentrations, particularly benzene, and adverse health risks in this study population. 

An important limitation of our study was our inability to assess VOC concentrations in Ogale’s groundwater system due to logistical constraints. The elevated levels of benzene and naphthalene found in Ogale’s groundwater system as described by the UNEP report suggest that the VOCs observed in indoor air in this study are likely present in the soil and groundwater in Ogale. This means that there might be additional health risks conferred from these other exposure sources, especially from Ogale’s groundwater system. This limitation warrants total exposure assessments to characterize VOC concentrations in outdoor spaces, soil and ground water, and local human activity patterns. Personal and biomarker monitoring is also suggested to obtain individual-level exposures for epidemiologic studies [37]. These assessments would add to the findings presented in this study and provide a more robust characterization of current VOC exposure and associated health risks present in the Niger Delta. 

## 5. Conclusions

In this work, we present evidence of elevated indoor air VOC concentrations and adverse predicted health risks that suggest a need for future and more extensive studies to make meaningful recommendations for intervention. This study alone does not indicate a need for, but provides further support for, UNEP’s recommendation for comprehensive monitoring in the Niger Delta and the establishment of a health registry, medical surveillance, and a prospective cohort study in Ogale [5]. Additional studies of indoor VOCs in Sub-Saharan Africa and other developing countries experiencing poor air quality are needed. A greater understanding of the sources of VOC exposures and the associated health risks in this environment will be beneficial to public health investigations of environmental contamination in Sub-Saharan Africa. 

## Figures and Tables

**Table 1:** Indoor VOC concentrations across all sampling locations.

VOC (µg/m^3^)	CAS Number	Detection Frequency (%)	Mean Indoor Concentrations	Median Indoor Concentrations	Standard Deviation	Min	Max
Benzene	71-43-2	100	25.7	16.1	23.2	9.1	105.4
Carbon tetrachloride	56-23-5	100	1.0	1.0	0.2	0.6	1.5
d-Limonene	5989-27-5	100	1.8	1.4	1.2	0.2	4.2
Dimethylfuran, 2,5-	625-86-5	50	0.1	0.3 × 10^1^	0.2	0.0	0.7
Dioxane, 1,4-	123-91-1	95	0.2	0.1	0.1	0.0	0.4
Ethylbenzene	100-41-4	100	5.2	2.5	10.4	1.0	48.2
Isopropylbenzene	98-82-8	100	0.4	0.2	0.6	0.1	3.0
m,p-Xylene ^a^	179601-23-1	100	10.7	6.2	15.7	2.4	74.2
Naphthalene	91-20-3	100	7.6	2.2	13.8	0.7	60.0
o-Xylene	95-47-6	100	4.2	2.4	6.5	0.9	30.5
p-Dichlorobenzene	106-46-7	100	5.1	0.4	12.1	0.2	42.0
p-Isopropyltoluene	99-87-6	100	0.2	0.1	0.1	0.1	0.4
Propylbenzene	103-65-1	100	0.8	0.4	1.3	0.2	5.9
sec-Butylbenzene	135-98-8	100	0.2	0.1	0.4	0.0	1.8
Styrene	100-42-5	100	1.8	0.9	4.0	0.4	18.7
Trimethylbenzene, 1,2,3-	526-73-8	100	1.7	1.1	1.6	0.5	6.8
Trimethylbenzene, 1,2,4-	95-63-6	100	4.0	2.7	4.1	0.9	18.2
Trimethylbenzene, 1,3,5-	108-67-8	100	1.6	0.9	1.7	0.4	7.9
Toluene	108-88-3	100	21.9	12.5	34.7	6.2	163.4
Xylenes (mixed) ^b^	1330-20-7	100	14.8	8.8	22.2	3.3	104.7

Abbreviations: CAS Chemical abstract service. ^a^ m- and p-xylene could not be chromatographically separated, and were treated as one compound; ^b^ Mixed xylene was the sum of m-, p-, and o-xylenes.

**Table 2:** VOCs and Estimated Non-Cancer Hazards and Cancer Risks.

VOC	Reference Concentration [16] (μg/m^3^)	Hazard Quotient	IUR [17] (1/10^6^) (μg/m^3^)^−1^	Cancer Risk	% Total Cancer Risk	Predicted Organ System(s) Impacted [16]
Benzene	3 × 10^1^	9 × 10^−1^	8 × 10^−6^	**2 × 10^−4^**	38	Immune
Carbon tetrachloride	1 × 10^2^	1 × 10^−2^	6 × 10^−6^	**6 × 10^−6^**	1.1	Hepatic
Dioxane, 1,4-	3 × 10^1^	7 × 10^−3^	5 ×10^−6^	1 × 10^−6^	0.1	Nervous, Respiratory
Ethylbenzene	1 × 10^3^	5 × 10^−3^	3 × 10^−6^	**1 × 10^−5^**	2.5	Developmental
Naphthalene	3 × 10^0^	3 × 10^0^	3 × 10^−5^	**3 × 10^−4^**	48	Nervous, Respiratory
p-Dichlorobenzene	8 × 10^2^	6 × 10^−3^	1 × 10^−5^	**6 × 10^−5^**	10	Hepatic
Styrene	1 × 10^3^	2 × 10^−3^	-	-	-	Nervous
Toluene	5 × 10^3^	4 × 10^−3^	-	-	-	Nervous
Trimethylbenzene, 1,2,4-	6 × 10^1^	7 × 10^−2^	-	-	-	Nervous
Xylenes (mixed)	1 × 10^2^	1 × 10^−1^	-	-	-	Nervous

Bold indicates a lifetime inhalation cancer risk >1 × 10^−6^; [-]: Inhalation unit rate not available for that VOC.

**Table 3:** Univariate Logistic Regression Estimates for the Association between Select Indoor VOCs and Self-Reported Health Symptoms.

VOC	Neurologic Symptoms OR (95% CI)	Skin Irritation OR (95% CI)	Eye Irritation OR (95% CI)	Throat Irritation OR (95% CI)	Aplastic Anemia OR (95% CI)	Gastrointestinal Symptoms OR (95% CI)
Benzene	0.9 (0.9, 1.0)	0.9 (0.9, 1.0)	1.0 (1.0, 1.1)	1.0 (0.9, 1.1)	1.0 (1.0,1.1)	1.1 (1.0, 1.2)
Carbon tetrachloride	-	-	-	-	-	-
Dioxane, 1,4-	-	-	-	-	-	-
Ethylbenzene	0.9 (0.7, 1.3)	0.8 (0.4, 1.6)	1.1 (0.9, 1.3)	1.0 (0.8, 1.3)	1.1 (0.9, 1.5)	1.1 (0.9, 1.4)
Naphthalene	1.0 (0.9, 1.1)	0.8 (0.4, 1.4)	0.9 (0.8, 1.1)	0.9 (0.6, 1.5)	0.9 (0.6, 1.4)	0.9 (0.6, 1.4)
p-Dichlorobenzene	0.9 (0.7, 1.2)	-	-	1.0 (0.8, 1.2)	-	-
Styrene	0.6 (0.1, 4.7)	0.5 (0.1, 6.0)	1.4 (0.6, 3.1)	0.9 (0.3, 3.1)	1.5 (0.5, 4.7)	1.4 (0.7, 2.9)
Toluene	1.0 (0.9, 1.1)	0.9 (0.8, 1.1)	1.1 (1.0, 1.1)	1.1 (1.0, 1.1)	1.0 (1.0, 1.1)	1.0 (1.0, 1.1)
Trimethylbenzene, 1,2,4-	1.0 (0.7, 1.2)	0.9 (0.6, 1.3)	1.2 (0.9, 1.6)	1.0 (0.7, 1.6)	1.3 (1.0, 1.8)	1.3 (1.0, 1.8)
Xylenes (mixed)	1.0 (0.9, 1.1)	1.0 (0.8, 1.1)	1.0 (1.0, 1.1)	1.0 (0.9, 1,1)	1.1 (1.0, 1.2)	1.1 (1.0, 1,1)

Abbreviations: OR Odds ratio, CI Confidence interval; [-]: Odds ratios were not estimated due to low event frequency for that VOC.
